# Association of high-sensitivity C-reactive protein and lipoprotein-associated phospholipase A_2_ with coronary artery lesion severity

**DOI:** 10.3389/fphys.2026.1734055

**Published:** 2026-06-22

**Authors:** Shi-Ning Luo, Xiang-Yi Guo, Qing-Li Liu, Zhi-Juan Li

**Affiliations:** Department of Cardiology, The First Affiliated Hospital, and College of Clinical Medicine of Henan University of Science and Technology, Luoyang, China

**Keywords:** coronary atherosclerotic heart disease, Gensini score, high-sensitivity C-reactive protein, Hs-CRP, lipoprotein-associated phospholipase A2, Lp-PLA2

## Abstract

**Objective:**

To evaluate the associations of high-sensitivity C-reactive protein (hs-CRP) and lipoprotein-associated phospholipase A_2_ (Lp-PLA_2_) with coronary lesion severity in patients undergoing coronary angiography, and to clarify their respective clinical utility.

**Methods:**

A total of 438 hospitalized patients who underwent coronary angiography in the Department of Cardiology of the First Affiliated Hospital of Henan University of Science & Technology between April 2023 and April 2024 were retrospectively analyzed. Based on the extent of coronary artery stenosis, participants were divided into a CHD group (stenosis ≥50%, n = 329) and a control group (stenosis <50%, n = 109). General clinical data and laboratory indicators, including Lp-PLA_2_ and hs-CRP levels, were collected and compared between groups. Independent risk factors for CHD were analyzed using binary logistic regression. Receiver operating characteristic curve analysis was conducted to evaluate the diagnostic discrimination (area under the curve) of each biomarker for concurrent assessment of lesion severity. Statistical analysis was performed using SPSS 27.0. Continuous data were compared using t-tests or nonparametric tests; categorical data were compared with χ² tests. Correlation and multivariate logistic regression analyses were conducted, and ROC curves assessed biomarker diagnostic discriminatory utility. p<0.05 was considered statistically significant.

**Results:**

Serum levels of both Lp-PLA_2_ and hs-CRP demonstrated a significant association with the severity of coronary artery lesions. Lp-PLA_2_ levels increased in correlation with the number of affected vessels, reaching the highest levels in individuals with three-vessel disease. Conversely, hs-CRP levels were highest among those with two-vessel disease. Participants with acute myocardial infarction exhibited higher levels of both biomarkers compared to those with stable or unstable angina (all *p* < 0.05). Lp-PLA_2_ demonstrated excellent diagnostic discrimination for CHD (AUC = 0.900) and severe coronary lesions (AUC = 0.952). While the incremental gain of combined hs-CRP assessment was modest (ΔAUC = 0.007, p = 0.427), the dual-marker approach provides complementary inflammatory information for risk stratification.

**Conclusion:**

The combined evaluation of Lp-PLA_2_ and hs-CRP provides clinically meaningful diagnostic discriminatory information regarding the severity of coronary artery lesions. This dual-marker approach was associated with coronary lesion severity in individuals undergoing coronary angiography in this retrospective cohort.

## 1 Introduction

Cardiovascular disease (CVD) remains one of the leading causes of mortality and disability worldwide. Coronary heart disease (CHD), the most prevalent form of CVD, continues to exhibit a rising incidence, primarily attributable to population aging and lifestyle modifications. According to the China Cardiovascular Health and Disease Report 2023 Summary (Summary of the 2023 report on cardiovascular health and diseases in China, 2024), there are currently 330 million individuals with CVD in China, among whom 11.39 million are diagnosed with coronary atherosclerotic heart disease, and mortality rates continue to rise, representing a considerable challenge to the public health system. In central China, alongside accelerated population aging and the widespread adoption of urbanized lifestyles, the prevention and control of atherosclerotic cardiovascular diseases face increasing pressure. As a regional cardiovascular diagnosis and treatment center, our hospital annually attends to a large number of patients with suspected coronary heart disease. In clinical practice, identifying non-invasive biomarkers associated with coronary lesion severity may complement clinical assessment in symptomatic patients referred for coronary angiography.

Atherosclerosis (AS) constitutes the primary pathological process underlying CHD and is characterized by endothelial injury, lipid accumulation, and chronic vascular inflammation (Rahman and Woollard, 2017; Liang and Wang, 2021). Increasing evidence supports the role of inflammation in both the initiation and progression of AS (Wolf and Ley, 2019; Kong et al., 2022). In this context, inflammatory biomarkers like lipoprotein-associated phospholipase A_2_ (Lp-PLA_2_) and high-sensitivity C-reactive protein (hs-CRP) have gained attention due to their roles in atherogenic pathways and their influence on plaque stability. Lp-PLA_2_ primarily binds to low-density lipoprotein (LDL) and participates in the oxidative modification of LDL within the vascular wall. This process contributes to endothelial injury, stimulates immune cell infiltration and cytokine release, thereby exacerbating vascular inflammation, increasing plaque instability, and accelerating the formation of atherosclerotic plaques. Consequently, Lp-PLA_2_ plays a significant role in promoting vascular inflammation and the development of atherosclerotic lesions (Yang et al., 2006; De Stefano et al., 2019; Khatana et al., 2020; Diaconu et al., 2021). And hs-CRP is not a direct causative factor of cardiovascular disease but rather serves as a biomarker of systemic low-grade inflammation mediated by cytokines such as interleukin-6 (IL-6) and interleukin-1β (IL-1β). These cytokines participate in the progression of atherosclerosis by promoting procoagulant activity, enhancing adhesion of monocytes and leukocytes to vascular endothelial cells, and stimulating proliferation of vascular smooth muscle cells (Ridker et al., 2017).

Although coronary angiography (CAG) remains the gold standard for diagnosing CHD and assessing the severity of vascular lesions, its invasive nature, high cost, and potential risks restrict its broader clinical utility. Consequently, there is growing interest in the development of non-invasive, efficient, and widely applicable serological markers for CHD assessment. Although numerous studies have examined the associations of Lp-PLA_2_ and hs-CRP as independent biomarkers of CHD, studies into their combined value, particularly in relation to lesion severity, remain limited.

In the present study, data from 438 patients admitted to the Department of Cardiology of the First Affiliated Hospital of Henan University of Science & Technology between April 2023 and April 2024 were retrospectively analyzed to examine the correlations between Lp-PLA_2_ and hs-CRP levels with the presence and severity of CHD, as determined by the Gensini scoring system. The study aimed to evaluate the clinical utility of these biomarkers, individually and in combination, for assessing concurrent disease severity and refining risk stratification among symptomatic patients undergoing angiography.

## 2 Data and methods

### 2.1 Study participants

A total of 543 patients who were hospitalized in the Department of Cardiology and underwent CAG between April 2023 and April 2024 were retrospectively analyzed. Following the application of inclusion and exclusion criteria, 438 patients fulfilled the eligibility requirements and were included in the final analysis. Based on CAG findings, 329 patients who met the diagnostic criteria for CHD were assigned to the CHD group, while 109 patients whose imaging results did not meet the diagnostic criteria were assigned to the control group. It is important to clarify that the control group comprised symptomatic patients who underwent clinically indicated coronary angiography but were found to have non-obstructive coronary arteries (stenosis <50%), rather than healthy asymptomatic individuals. This reflects real-world clinical practice where biomarkers are used to evaluate the need for invasive angiography. Among the 109 control patients, 73 (67.0%) had completely normal coronary arteries (Gensini score = 0), while 36 (33.0%) exhibited minimal atherosclerotic changes with Gensini scores ranging from 1 to 6. These patients may have non-cardiac chest pain, mild coronary atherosclerosis, or other causes of symptoms (e.g., microvascular angina, esophageal spasm).

The study protocol was reviewed and approved by the Ethics Committee of the First Affiliated Hospital of Henan University of Science & Technology (Ethics Approval No.: K-2025-B007).

### 2.2 Inclusion and exclusion criteria

#### 2.2.1 Inclusion criteria

Participants were included if they met all of the following conditions:

1. Admission with typical symptoms such as chest tightness or chest pain, with a preliminary suspicion of coronary atherosclerotic heart disease.
2. Underwent relevant auxiliary examinations after admission, were fully informed of the details and risks associated with CAG, provided written informed consent for the procedure, and subsequently underwent CAG in the hospital.
3. Possessed CAG imaging results that met post-processing quality standards.
4. Had complete medical records, including general clinical data, medical history, and comprehensive laboratory and auxiliary examination data.

#### 2.2.2 Exclusion criteria

Participants were excluded based on any of the following conditions:

1. History of thrombolytic therapy, coronary artery bypass grafting, or percutaneous coronary intervention performed either in the current hospital or in other institutions.
2. Diagnosis of arrhythmia, cardiomyopathy, heart failure, congenital heart disease, rheumatic heart disease, or other CVDs associated with myocardial injury.
3. Presence of infectious diseases or development of severe infections after admission. Patients with elevated white blood cell count (>10×10^9^/L) or fever (>38°C) at admission were additionally reviewed to exclude occult infection.
4. Diagnosed hematological disorders, malignant tumors, immune system disorders, or current long-term use of corticosteroids or lipid-lowering medications.
5. Severe hepatic or renal dysfunction, defined as serum transaminase levels greater than twice the upper limit of normal or serum creatinine >442 μmol/L.
6. Incomplete clinical or laboratory data essential for the analysis.

### 2.3 Data collection

Demographic and clinical data including sex, age, body mass index (BMI), history of hypertension, history of diabetes, smoking status, and alcohol consumption history, were obtained from the hospital’s electronic medical record system.

Smoking status was categorized as current (active smoking within past 6 months), former (quit >6 months ago), or never. Pack-years were calculated for current and former smokers. Diabetes mellitus was defined as fasting glucose ≥7.0 mmol/L, HbA1c ≥6.5%, or current use of antidiabetic medications. Type 1 and Type 2 diabetes were not distinguished in this analysis. Hypertension was defined as systolic BP ≥140 mmHg, diastolic BP ≥90 mmHg on at least two occasions, or current use of antihypertensive medications. BP levels were recorded as the average of three seated measurements.

Laboratory parameters were obtained from peripheral blood samples collected within 24 hours of admission and included Lp-PLA_2_, hs-CRP, total cholesterol (TC), triglycerides (TG), high-density lipoprotein cholesterol (HDL-C), low-density lipoprotein cholesterol (LDL-C), apolipoprotein B (Apo-B), apolipoprotein A (Apo-A), lipoprotein(a) ^[Lp(a)]^, and homocysteine (Hcy). Coronary angiography results for all enrolled participants were collected and systematically evaluated.

The severity of coronary artery lesions was assessed using the Gensini scoring system, based on angiographic findings. Coronary angiograms were reviewed independently by two experienced interventional cardiologists (X.Y.G. and Q.L.L.) who were blinded to biomarker results. The Gensini score was calculated according to the standard algorithm: severity factor × location factor. Inter-observer agreement was assessed using intraclass correlation coefficient (ICC = 0.94), indicating excellent reliability. Discrepancies were resolved by consensus with a third reviewer (Z.J.L.). The Gensini score exhibited a right-skewed distribution (skewness = 0.504, Shapiro-Wilk p < 0.001). To address this, we additionally categorized patients into tertiles (mild, moderate, severe) for ordinal logistic regression and subgroup comparisons (**Table 1**), while retaining the continuous Gensini score for primary multivariate linear regression to preserve statistical power.

**Table 1:** Comparison of clinical and biochemical parameters among groups with different lesion severities.

Variables	Mild (n = 83)	Moderate (n = 164)	Severe (n = 82)	Test value	P
Gender				0.729^a^	0.694
Male	55 (66.3)	102 (62.2)	55 (67. 1)		
Female	28 (33.7)	62 (37.8)	27 (32.9)		
Age	59.00 (53.00,64.00)	56.00 (46.25,62.00)	57.00 (46.00,63.00)	7.784^#^	0.020
BMI	26.12 (23.13,28.80)	25.35 (23.24,28.00)	25.72 (23.77,28.19)	2.074#	0.355
Smoking				0.892^a^	0.640
No	31 (37.3)	52 (31.7)	26 (31.7)		
Yes	52 (62.7)	112 (68.3)	56 (68.3)		
Alcohol consumption				0.741^a^	0.690
No	37 (44.6)	79 (48.2)	35 (42.7)		
Yes	46 (55.4)	85 (51.8)	47 (57.3)		
Hypertension				1.541^a^	0.463
No	45 (54.2)	76 (46.3)	38 (46.3)		
Yes	38 (45.8)	88 (53.7)	44 (53.7)		
Diabetes				1.232^a^	0.540
No	25 (30.1)	60 (36.6)	26 (31.7)		
Yes	58 (69.9)	104 (63.4)	56 (68.3)		
Lp-PLA_2_ (ng/mL)	137.00 (110.57,162.50)	171.02 (154.14,183.84)	183.36 (160.76, 207.49)	60.793^#^	<0.001
hs-CRP (mg/L)	1.90 (1.34,3.08)	3.78 (2.44,5.46)	4.72 (3.11,8.72)	59.041#	<0.001
TC	3.06 (2.65,3.62)	3.56 (2.84,3.93)	3.70 (3.24,4.23)	14.603#	<0.001
TG	1.51 (0.89,2.59)	3.32 (1.67,4.83)	2.77 (1.60,4.97)	35.921#	<0.001
HDL-C	1. 17 (0.91,1.58)	1.57 (1.06,2.24)	1.48 (1.05,2.15)	13.870#	<0.001
LDL-C	2.70 (1.93,3.77)	3.05 (2.34,3.55)	2.98 (2.31,3.55)	0.538#	0.764
Apo-A	1.08 (0.99,1.15)	1. 04 (0.97,1.11)	1. 04 (0.97,1.12)	5.262#	0.072
Apo-B	1.03 (0.93,1.12)	1.03 (0.93,1.09)	1.03 (0.93,1.11)	0.541#	0.763
Lp(a)	23.77 (18.41,27.93)	23.53 (18.73,27.74)	23.98 (19.27,30.10)	1.268#	0.530
Hcy	21.00 (18.0,23.0)	20.00 (19.0,22.75)	21.00 (18.0,23.0)	0.130#	0.937

^#^Mann–Whitney U test; ^a^chi-squared test.

*Blood sample collection and processing:* Fasting venous blood samples (minimum 8 hours fasting) were collected from all participants on the morning of coronary angiography, prior to the administration of heparin, statins, antiplatelet agents, and prior to the procedure. For patients presenting with acute coronary syndromes (ACS), blood samples were drawn immediately upon hospital admission within 6–24 hours after symptom onset, before therapeutic intervention. Given that hs-CRP levels rise acutely during myocardial infarction as part of the acute-phase response, we acknowledge that hs-CRP values in AMI patients may reflect both chronic vascular inflammation and acute myocardial injury. Blood was collected into serum separator tubes, allowed to clot for 30 minutes at room temperature, and centrifuged at 3000 rpm for 15 minutes. The separated serum was either analyzed immediately (within 4 hours) or stored at -70 °C in aliquots and thawed only once prior to analysis (within 4 weeks).

*High-sensitivity C-reactive protein (hs-CRP):* Serum hs-CRP levels were measured using a particle-enhanced immunoturbidimetric assay. The assay kit was the hs-CRP Latex Reagent Kit (Maccura Biotechnology Co., Ltd., Chengdu, China). Measurements were performed on the PA 200 fully automated specific protein analyzer (Maccura Biotechnology Co., Ltd., Chengdu, China). The detection limit was 0.5 mg/L, with a linear range of 0.5–420.0 mg/L. The intra-assay coefficient of variation (CV) was < 10.0%, and the inter-assay CV was < 15.0%. The reference range for hs-CRP was < 3.0 mg/L.

*Lipoprotein-associated phospholipase A_2_ (Lp-PLA_2_):* Serum Lp-PLA_2_ levels were measured using a quantitative immunoassay method. The assay kit was the Lipoprotein-Associated Phospholipase A2 Detection Kit (Beijing Hotgen Biotech Co., Ltd., Beijing, China). Measurements were performed on the QMT8000 ImmunoQuant Analyzer (Wuhan Mingde Biotechnology Co., Ltd., Wuhan, China). The assay measured Lp-PLA_2_ mass concentration (ng/mL). The measuring range was 5–800 ng/mL, with a reference range of < 175 ng/mL. The intra-assay CV was < 10%, and the inter-assay CV was < 15%. All measurements were performed in duplicate according to the manufacturer’s instructions, and the average value was used for analysis.

*Quality control:* Commercial quality control materials with known concentrations were run in each batch to ensure assay accuracy. Samples with CV exceeding 15% between duplicate measurements were reanalyzed. All biomarker measurements were performed by trained laboratory technicians blinded to the clinical and angiographic data.

### 2.4 Grouping method

Based on coronary angiography results and the diagnostic criteria for CHD, the 438 eligible participants were categorized into two primary groups: the CHD group (*n* = 329) and the control group (*n* = 109).

**Figure 1:**
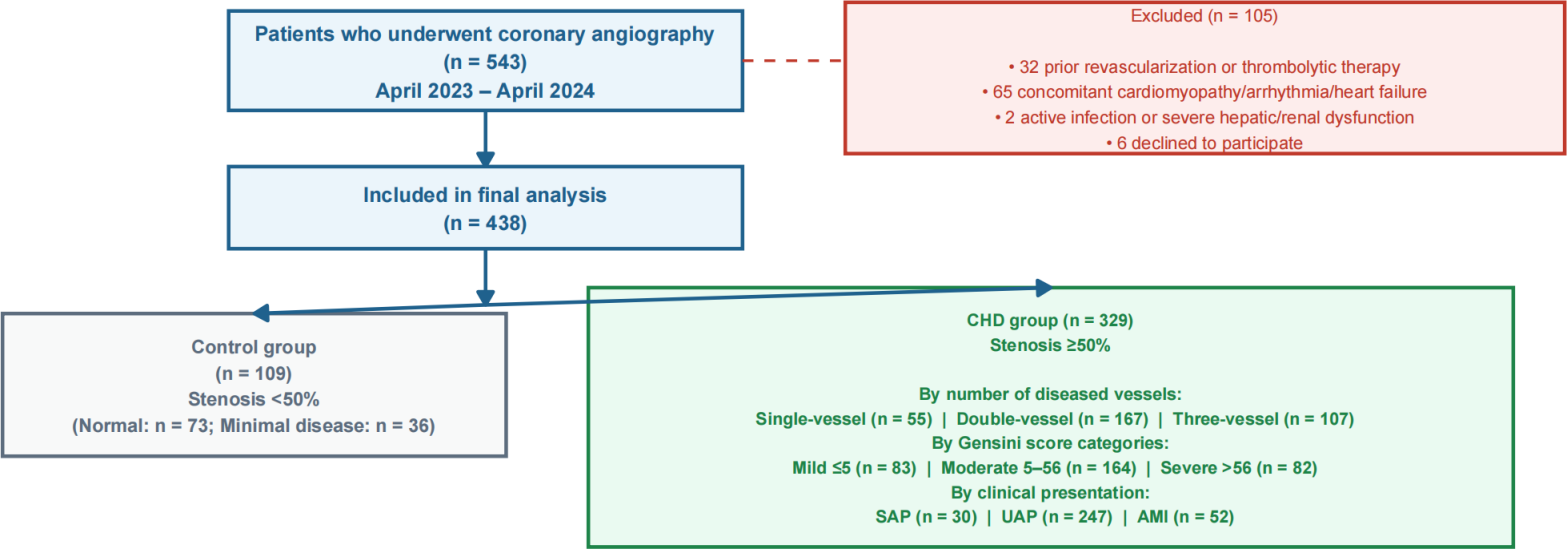
Study cohort selection and exclusion flowchart. Of 543 patients who underwent coronary angiography between April 2023 and April 2024, 105 were excluded based on prespecified criteria: 32 with prior revascularization or thrombolytic therapy, 65 with concomitant cardiomyopathy/arrhythmia/heart failure, 2 with active infection or severe hepatic/renal dysfunction and 6 declined to participate. The remaining 438 participants were categorized into a control group (n = 109, stenosis <50%) and a CHD group (n = 329, stenosis ≥50%), with further stratification by number of diseased vessels, Gensini score categories, and clinical presentation.

Within the CHD group, participants were further stratified according to the number of affected coronary vessels into three subgroups: single-vessel disease (*n* = 55), double-vessel disease (*n* = 167), and three-vessel disease (*n* = 107). The severity of coronary artery lesions was quantified using the Gensini score derived from angiographic findings. Based on Gensini score tertile-based cutoffs, patients were classified into the mild lesion group (score ≤ 5, *n* = 83), the moderate lesion group (5 < score ≤ 56, *n* = 164), and the severe lesion group (score > 56, *n* = 82). Additionally, based on clinical presentation, the CHD group was divided into three subgroups: stable angina pectoris (SAP, *n* = 30), unstable angina pectoris (UAP, *n* = 247), and acute myocardial infarction (AMI, *n* = 52), see **Figure 1**.

### 2.5 Statistical analysis

#### 2.5.1 General statistical description and group comparisons

Data were organized using Microsoft Excel and analyzed using SPSS version 27.0 (IBM Corp., Armonk, NY, USA). Continuous variables were tested for normality using the Shapiro-Wilk test and visual inspection of Q-Q plots. Normally distributed data are presented as mean ± standard deviation (SD) and were compared between two groups using the independent-samples t-test or among multiple groups using one-way analysis of variance (ANOVA) with Bonferroni *post-hoc* correction for pairwise comparisons. Non-normally distributed data are expressed as median (interquartile range [IQR]) and were compared using the Mann–Whitney U test (two groups) or the Kruskal–Wallis H test with Dunn-Bonferroni *post-hoc* adjustment (multiple groups). Categorical variables are expressed as frequencies (percentages) and were compared using Pearson’s chi-squared test or Fisher’s exact test where appropriate.

#### 2.5.2 Correlation analyses

Correlation analyses were performed using Pearson’s correlation for normally distributed variables and Spearman’s rank correlation for non-normally distributed variables.

#### 2.5.3 Regression modeling

For binary logistic regression, variables demonstrating univariate significance (p < 0.10) or established clinical relevance were entered simultaneously using forced entry (simultaneous entry) to avoid stepwise selection bias: age (continuous), sex (male/female), smoking status (yes/no), diabetes mellitus (yes/no), hypertension (yes/no), body mass index (continuous), total cholesterol (continuous), LDL-C (continuous), HDL-C (continuous). Apolipoprotein B, apolipoprotein A, triglycerides, lipoprotein(a), and homocysteine were excluded from the multivariate model due to quasi-complete separation caused by their strong correlations with other lipid parameters. The full model results, including non-significant variables, are presented in **Table 2**. Odds ratios for continuous biomarkers (Lp-PLA_2_ and hs-CRP) are reported per standard deviation (SD) increment to facilitate clinical interpretability, in addition to per-unit estimates. The linearity assumption for continuous variables was assessed using Box-Tidwell tests. Variance inflation factor (VIF) values were all <5.0, indicating no problematic multicollinearity.

**Table 2:** Results of binary logistic regression analysis of risk factors for CHD.

Variable	B	Se	Wald x2	P	OR value	95% confidence interval for OR	
						Lower limit	Upper limit
Constant	-14.966	3.232	21.442	<0.001			
Age	0.101	0.026	15.074	<0.001	1.106	1.051	1.164
Sex	0.346	0.502	0.476	0.490	1.414	0.528	3.783
Smoking	1.170	0.461	6.448	0.011	3.223	1.306	7.953
Diabetes	1.715	0.472	13.220	<0.001	5.559	2.205	14.015
Hypertension	0.120	0.467	0.066	0.798	1.127	0.451	2.817
BMI	0.125	0.084	2.220	0.136	1.133	0.961	1.336
TC	-0.942	0.228	17.041	<0.001	0.390	0.249	0.610
HDL-C	-0.797	0.337	5.589	0.018	0.451	0.233	0.873
LDL-C	1.444	0.288	25.084	<0.001	4.239	2.409	7.460
Lp-PLA_2_ (ng/mL)	0.047	0.007	46.517	<0.001	1.048 (per unit) 9.669 (per SD=48.33 ng/mL)	1.034	1.062
hs-CRP (mg/L)	0.426	0.148	8.317	0.004	1.531 (per unit) 8.895 (per SD=5.13 mg/L)	1.146	2.044

OR, odds ratio; CI, confidence interval. Variables excluded due to quasi-complete separation: apolipoprotein B, apolipoprotein A, triglycerides, lipoprotein(a), and homocysteine. Per-SD ORs were calculated by multiplying the regression coefficient by the SD of the respective variable and exponentiating.

#### 2.5.4 Collinearity diagnostics

We assessed multicollinearity using variance inflation factor (VIF) and tolerance values. Specifically examining the relationship between Lp-PLA_2_ and LDL-C (which are physiologically correlated), the VIF was well below the threshold of concern (VIF < 5.0). All other variables showed VIF values < 3.0, indicating no problematic multicollinearity.

#### 2.5.5 ROC analysis and model comparison

Receiver operating characteristic (ROC) curves were constructed to evaluate discriminatory performance. The combined biomarker model was constructed using binary logistic regression with both Lp-PLA_2_ and hs-CRP entered simultaneously as continuous variables. The predicted probability from this model was used as the test variable for ROC analysis. The logistic regression equation was:


$$\text{Logit}\;\left(\text{P}\right)\;=\;{\text{β}}_{0}\;+\;{\text{β}}_{1}\;\times \;\left(\text{standardized}\;{\text{Lp-PLA}}_{2}\right)\;+\;{\text{β}}_{2}\;\left(\text{standardized}\;\text{hs}-\text{CRP}\right)$$

where standardized values were calculated as (value - mean)/SD. The predicted probability P = exp(Logit)/(1+exp(Logit)).

No interaction term was included between Lp-PLA_2_ and hs-CRP as the interaction was not statistically significant (*p* = 0.34).

Two ROC analyses were performed to address distinct clinical questions. First, to evaluate the diagnostic discrimination of Lp-PLA_2_ and hs-CRP for CHD versus non-CHD, the full cohort (n = 438) was used, with CHD status (stenosis ≥50%) as the binary outcome. Second, to assess the ability of these biomarkers to identify severe coronary lesions, a secondary analysis compared patients with severe CHD (Gensini score >56, n = 82) against the control group (n = 109), excluding patients with intermediate lesion severity (5 < Gensini ≤56, n = 164) and mild CHD (Gensini ≤5, n = 83). This approach was chosen because the control group comprised symptomatic patients with non-obstructive coronary arteries, representing the clinical population in whom biomarker-related clinical assessment is most relevant.

#### 2.5.6 Missing data

Cases with missing values in any of the analyzed variables were excluded using listwise deletion (complete case analysis). The final analytical sample included 438 participants.

A two-tailed p value of < 0.05 was considered statistically significant for all analyses except where Bonferroni correction was applied for multiple comparisons.

## 3 Results

### 3.1 Comparison of general and clinical characteristics between the CHD and control groups

A total of 438 patients were included in this study, with 329 assigned to the CHD group and 109 to the control group. Patients in the CHD group were significantly older and had higher proportions of smoking history and diabetes compared with those in the control group (all *p* < 0.05). No significant differences were identified between the two groups in terms of sex, BMI, drinking history, or history of hypertension (all *p* > 0.05). Analysis of serological parameters demonstrated significant differences between the two groups in Lp-PLA_2_, hs-CRP, TC, TG, LDL-C, HDL-C, Apo-B, Lp(a), and Hcy levels (all *p* < 0.05). The CHD group exhibited higher levels of these parameters compared with the control group, whereas Apo-A levels were higher in the control group (*p* < 0.05) (**Table 3**).

**Table 3:** Comparison of demographic and clinical characteristics between the control group and the CHD group.

General characteristics	Control group (n = 109)	CHD group (n = 329)	Test value	P-value
Sex (%)			0. 431^a^	0.562
Male	74 (67.9)	212 (64.4)		
Female	35 (32.1)	117 (35.6)		
Age (years)	48.00 (41.0,52.5)	57.00 (49.0,63.0)	7.744#	<0.001
BMI	25.39 (23.15,28.18)	25.65 (23.46,28.27)	0.715^#^	0.475
Smoking history (%)			49.419^a^	<0.001
No	78 (71.6)	109 (33.1)		
Yes	31 (28.4)	220 (66.9)		
Alcohol consumption (%)			0.026^a^	0.912
No	51 (46.8)	151 (45.9)		
Yes	58 (53.2)	178 (54.1)		
History of hypertension (%)			2.938^a^	0.098
No	63 (57.8)	159 (48.3)		
Yes	46 (42.2)	170 (51.7)		
History of diabetes (%)			50.415^a^	<0.001
No	78 (72.9)	111 (33.7)		
Yes	29 (27.1)	218 (66.3)		
Serum biomarkers				
Lp-PLA_2_(ng/mL)	98.92 (77.34,114.34)	167.11 (145.81,186.42)	12.511#	<0.001
hs-CRP(mg/L)	2.41 (1.86,3.06)	3.45 (1.98,5.51)	5.542#	<0.001
TC(mmol/L)	4.25 (3.47,4.77)	3.49 (2.78,3.93)	-5.577#	<0.001
TG(mmol/L)	1.91 (1.37,2.50)	2.56 (1.45,4.68)	3.934#	<0.001
HDL-C(mmol/L)	1.61 (1.19,2.29)	1.43 (0.99, 2.10)	-2.269#	0.023
LDL-C(mmol/L)	1.87 (1.37,2.25)	2.99 (2.23,3.61)	9.620#	<0.001
Apo-A(g/L)	1.28 (1.19, 1.36)	1. 05 (0.97, 1.12)	-13.549#	<0.001
Apo-B(g/L)	0.78 (0.66,0.89)	1.03 (0.93, 1. 10)	11.946#	<0.001
Lp(a)	18.74 (14.00,22.80)	23.59 (18.73,28.23)	7.105#	<0.001
Hcy	12.00 (8.02,13.00)	21.00 (18.50,23.00)	14.983#	<0.001

^#^Mann–Whitney U test; ^a^chi-squared test.

Control group, patients with suspected CHD symptoms but angiographically <50% stenosis (non-obstructive CAD).

### 3.2 Multivariate logistic regression analysis of CHD risk factors

Twelve variables that demonstrated statistical significance in univariate analyses, including age, smoking status, and diabetes, were included in a binary logistic regression model. The results indicated that age (OR = 1.106, 95% CI: 1.051-1.164, p < 0.001), smoking (OR = 3.223, 95% CI: 1.306-7.953, p = 0.011), diabetes (OR = 5.559, 95% CI: 2.205-14.015, p < 0.001), LDL-C (OR = 4.239, 95% CI: 2.409-7.460, p < 0.001), Lp-PLA_2_ (OR = 1.048 per 1 ng/mL, 95% CI: 1.034-1.062, p < 0.001; OR = 9.669 per SD [48.33 ng/mL]), and hs-CRP (OR = 1.531 per 1 mg/L, 95% CI: 1.146-2.044, p = 0.004; OR = 8.895 per SD [5.13 mg/L]) were independent risk factors for coronary artery lesions. Additionally, total cholesterol (OR = 0.390, *p* < 0.001) and HDL-C (OR = 0.451, *p* = 0.018) were also significantly associated with CHD. Sex, hypertension, and BMI were not independently associated with CHD in the fully adjusted model (**Table 2**).

#### 3.2.1 Sensitivity analysis: age-matched subgroup

Given the significant age difference between the CHD and control groups (median 57.0 vs. 48.0 years, p < 0.001), we performed a 1:1 age-matched analysis (± 5 years) to assess whether the observed associations were independent of age. After matching, 90 pairs were identified with comparable ages (CHD: 46.6 ± 8.3 years vs. control: 46.5 ± 8.4 years, p = 0.944). In this matched cohort, Lp-PLA_2_ levels remained significantly higher in the CHD group compared with controls (160.4 [IQR: 134.2–183.7] vs. 96.2 [77.2–111.7] ng/mL, p < 0.001), as did hs-CRP levels (3.75 [1.81–4.94] vs. 2.38 [1.92–3.05] mg/L, p < 0.001). Paired Wilcoxon signed-rank tests confirmed significant differences for both biomarkers (both p < 0.001). These findings suggest that the associations between Lp-PLA_2_, hs-CRP, and CHD are not solely attributable to age differences between groups.

### 3.3 Analysis of factors among CHD subgroups

#### 3.3.1 Comparison of variables among CHD subgroups by number of diseased vessels

Subgroup analysis based on the number of significantly stenosed coronary vessels indicated no statistically significant differences in age, smoking status, alcohol consumption, history of hypertension, history of diabetes, TC, Lp(a), and Apo-B (*p* > 0.05). In contrast, significant differences were observed in Lp-PLA_2_, hs-CRP, TG, HDL-C, and LDL-C levels (*p* < 0.05), Sex, BMI, and Apo-A showed marginal differences.

Lp-PLA_2_ levels increased progressively with the number of diseased vessels, with the highest levels observed in the three-vessel group, followed by the double-vessel and single-vessel groups. hs-CRP levels were higher in the three-vessel and double-vessel groups compared with the single-vessel group. BMI was significantly higher in the three-vessel group compared to the double-vessel group on *post-hoc* analysis. TG and HDL-C reached the highest levels in the double-vessel group, whereas LDL-C was highest in the three-vessel group (all *p* < 0.05) (**Table 4**).

**Table 4:** Comparison of clinical and biochemical parameters among groups with different numbers of diseased vessels.

Single-vessel (n = 55)		Double-vessel (n = 167)	Three-vessel (n = 107)	Test value	P
Sex				5.251a	0.072
Male	37 (67.3)	98 (58.7)	77 (72.0)		
Female	18 (32.7)	69 (41.3)	30 (28.0)		
Age	59.00 (51.0,63.0)	56.00 (47.0,61.0)	58.00 (51.0,63.0)	4.966#	0.084
BMI	25.43 (22.95, 28.30)	25.22 (23.23,27.98)	26.78 (23.87,28.58)	5.903#	0.052
Smoking				1.559^a^	0.459
No	20 (36.4)	50 (29.9)	39 (36.4)		
Yes	35 (63.6)	117 (70.1)	68 (63.6)		
Alcohol consumption				0.726^a^	0.696
No	28(50.9)	74 (44.3)	49(45.8)		
Yes	27(49. 1)	93 (55.7)	58(54.2)		
History of hypertension				1.612^a^	0.447
No	29 (52.7)	75 (44.9)	55 (51.4)		
Yes	26 (47.3)	92 (55.1)	52(48.6)		
History of diabetes				3.826^a^	0.148
No	14 (25.5)	54 (32.3)	43 (40.2)		
Yes	41 (74.6)	113 (67.7)	64 (59.8)		
Lp-PLA_2_ (ng/mL)	156.00 (119.30, 172.75)	164.91 (145.60, 182.55)	177.82 (154.09, 204.16)	17.803#	<0.001
hs-CRP (mg/L)	2.45 (1.34,3.88)	4.21 (2.41,6.39)	3.22 (1.97,4.96)	16.701#	<0.001
TC	3.18 (2.38,3.86)	3.53 (2.90,3.91)	3.56 (2.65,4.00)	2.623#	0.269
TG	1.56 (1.09,3.41)	3.30 (1.67,4.97)	2.39 (1.36,3.70)	20.411#	<0.001
HDL-C	1.09 (0.85, 1.68)	1.64 (1. 14,2. 28)	1.26 (0.96, 1.91)	18.294#	<0.001
LDL-C	2.39 (1.86,3.49)	3.00 (2.36,3.43)	3.30 (2.30,3.79)	7.521#	0.023
Apo-A	1. 04 (0.96, 1.15)	1. 07 (1. 00, 1.13)	1. 03 (0.95, 1.10)	5.633#	0.060
Apo-B	1.03 (0.94, 1. 13)	1.03 (0.94, 1. 11)	1.02 (0.92, 1. 09)	1.603#	0. 449
Lp(a)	24.16 (18.92,28.39)	23.48 (18. 62,28.98)	23.35 (19.02,27.76)	0.693#	0.707
Hcy	20.00 (18.75,22.28)	21.00 (18.00,23.00)	20.00 (18.00,22.00)	1.274#	0.529

^#^Mann–Whitney U test; ^a^chi-squared test.

Notably, hs-CRP levels peaked in the double-vessel disease group rather than the three-vessel group. This unexpected pattern may be attributed to the higher proportion of ACS presentations in the double-vessel group compared to the three-vessel group, reflecting the acute inflammatory response associated with unstable plaques. Additionally, the timing of blood sampling relative to symptom onset may have influenced hs-CRP levels, as patients with three-vessel disease may have had more chronic, stable presentations with lower acute-phase reactants.

#### 3.3.2 Comparison of variables among CHD subgroups by clinical type

When stratified by clinical type, significant differences were identified in Lp-PLA_2_, hs-CRP, and HDL-C levels among the SAP, UAP, and AMI subgroups (all *p* < 0.001). The AMI subgroup exhibited significantly higher Lp-PLA_2_ and hs-CRP levels compared with both the SAP and UAP subgroups (all *p* < 0.001). Notably, hs-CRP levels were markedly elevated in the AMI subgroup (median 8.69 mg/L, IQR: 4.96-11.50) compared with the UAP (3.20 mg/L, IQR: 1.96-4.69) and SAP (1.83 mg/L, IQR: 1.21-3.25) subgroups. Additionally, the UAP subgroup demonstrated significantly higher Lp-PLA_2_ and HDL-C levels compared with the SAP subgroup (*p* < 0.001), whereas the difference between UAP and AMI in HDL-C was not statistically significant after Bonferroni correction (**Table 5**).

**Table 5:** Comparison of clinical and biochemical parameters among different CHD subtypes.

Variables	SAP(n=30)	UAP(n=247)	AMI(n=52)	Test Value	*P*
Sex				5.284^a^	0.071
Male	15 (50.0)	158 (64.0)	39 (75.0)		
Female	15 (50.0)	89 (36.0)	13 (25.0)		
Age	60.00 (49.00,66.25)	56.00 (49.00,62.00)	58.00 (52.00,67.00)	4.019#	0.134
BMI	25.91 (24.13,28.70)	25.50 (23.13,28.15)	25.72 (23.75,28.60)	2.629#	0.269
Smoking				1.733^a^	0.420
No	12(40.0)	77 (31.2)	20 (38.5)		
Yes	18 (60.0)	170 (68.8)	32 (61.5)		
Alcohol consumption				1.172^a^	0.556
No	11 (36.7)	115 (46.6)	25 (48.1)		
Yes	19 (63.3)	132 (53.4)	27 (51.9)		
History of hypertension				4.994^a^	0.082
No	19 (63.3)	111 (44.9)	29 (55.1)		
Yes	11 (36. 7)	136 (55.8)	23 (44.2)		
History of diabetes				3.193^a^	0.203
No	7 (23.3)	82 (33.2)	22 (42.3)		
Yes	23 (76.7)	165 (66.8)	30 (57.7)		
Lp-PLA_2_ (ng/mL)	135.75 (109.15,156.80)	168.72 (149.70,183.04)	183.92 (156.03,222.29)	25.380#	<0.001
hs-CRP (mg/L)	1.83 (1.21,3.25)	3.20 (1.96,4.69)	8.69 (4.96,11.50)	76.667#	<0.001
TC	3.62 (2.94,4.56)	3.47 (2.67,3.90)	3.59 (3.12,3.98)	3.632#	0.163
TG	2.16 (1.29,3.35)	2.67 (1.47,4.71)	2.15 (1.20,4.71)	2.336#	0.311
HDL-C	0.97 (0.68,1.18)	1.53 (1.05,2.18)	1.26 (1.04,1.94)	23.089#	<0.001
LDL-C	2.88 (1.83,3.69)	3.02 (2.30,3.59)	2.93 (2.14,3.56)	0.505#	0.777
Apo-A	1.07 (0.96,1.15)	1.06 (0.98,1.13)	1.03 (0.93,1.09)	6.356#	0.042
Apo-B	1.06 (0.96,1.12)	1.02 (0.93,1.09)	1.03 (0.93,1.11)	2.014#	0.365
Lp(a)	25.87 (17.82,28.61)	23.48 (18.72,28.19)	23.53 (18.95,28.47)	1.259#	0.533
Hcy	20.50 (17.00,23.00)	21.00 (18.75,23.00)	20.00 (19.00,22.00)	0.575#	0.750

^#^Mann–Whitney U test; ^a^chi-squared test.

##### 3.3.2.1 Sensitivity analysis: exclusion of AMI patients

To address the concern that elevated hs-CRP in AMI patients may reflect acute-phase inflammation rather than chronic disease burden, we conducted a sensitivity analysis excluding AMI patients (n = 52). In the remaining cohort comprising control subjects (n = 109) and patients with SAP or UAP (n = 277), both Lp-PLA_2_ and hs-CRP remained significantly higher in the CHD group compared with controls (Lp-PLA_2_: 167.11 [145.81-186.42] vs. 98.92 [77.34-114.34] ng/mL, *p* < 0.001; hs-CRP: 3.45 [1.98-5.51] vs. 2.41 [1.86-3.06] mg/L, *p* < 0.001). In multivariate logistic regression, both biomarkers retained independent significance. These findings indicate that the associations between Lp-PLA_2_, hs-CRP, and coronary lesion presence are not driven solely by the acute inflammatory response in AMI.

#### 3.3.3 Comparison of variables among CHD subgroups by lesion severity

Among the 329 patients in the CHD group, lesion severity was stratified using Gensini score categories based on tertile-derived cutoffs: mild (score ≤ 5, *n* = 83), moderate (5 < score ≤ 56, *n* = 164), and severe (score > 56, *n* = 82). The cohort included 64.4% males and 35.6% females. Comparisons among the groups indicated significant differences in Lp-PLA_2_, hs-CRP, TC, TG, and HDL-C levels (all p < 0.05). Age was also significantly higher in the mild group compared with the moderate group (*p* = 0.005). No significant differences were observed in BMI, LDL-C, Apo-A, Apo-B, Lp(a), or Hcy levels among the three severity groups. Increasing lesion severity was associated with significantly higher levels of Lp-PLA_2_, hs-CRP, and TG (**Table 1**).

TG levels in the mild group were lower than those observed in the moderate and severe groups (*p* < 0.001). HDL-C levels were significantly higher in the moderate group compared with the mild group (*p* < 0.001); however, the difference between the moderate and severe groups was not statistically significant (*p* = 0.583), and no significant correlation was observed between HDL-C and the Gensini score in Spearman analysis (r = 0.111, *p* = 0.044). Apo-A levels showed marginal differences among groups (*p* = 0.072) but did not reach statistical significance after Bonferroni correction.

### 3.4 Spearman correlation analysis between risk factors and Gensini score

Among patients with coronary artery lesions, Spearman’s correlation analysis demonstrated significant positive correlations between the Gensini score and Lp-PLA2 (r = 0.431, *p* < 0.001), hs-CRP (r = 0.417, *p* < 0.001), TC (r = 0.215, *p* < 0.001), and TG.

(r = 0.254, *p* < 0.001). HDL-C showed a weak positive correlation (r = 0.111, *p* = 0.044). LDL-C, Apo-A, Apo-B, Lp(a), and Hcy did not demonstrate significant correlations with the Gensini score.

### 3.5 Multivariate linear regression analysis

A multivariate linear regression model was constructed using the Gensini score as the dependent variable among the 329 patients with coronary artery lesions. Independent variables included Lp-PLA2, hs-CRP, TG, LDL-C, and Apo-A. The model explained 22.6% of the variance in Gensini scores (R² = 0.226, adjusted R² = 0.214, F = 18.83, p < 0.001). Regression results indicated that Lp-PLA2 (B = 0.231, 95% CI: 0.161–0.301, p < 0.001), hs-CRP (B = 1.070, 95% CI: 0.580–1.561, p < 0.001), and Apo-A (B = -32.342, 95% CI: -56.475 to -8.210, p = 0.009) were significantly associated with the Gensini score. TG (B = 1.011, p = 0.102) and LDL-C (B = 0.743, p = 0.615) did not reach statistical significance in the fully adjusted model. We note elevated variance inflation factor (VIF) values for Lp-PLA2 (VIF = 15.8), LDL-C (VIF = 10.1), and Apo-A (VIF = 20.1), reflecting physiological correlations among lipid parameters; this is acknowledged as a model limitation (**Table 6**).

**Table 6:** Multivariate linear regression analysis.

Variables	B (95%CI)	*P*
Lp-PLA_2_ (ng/mL)		
Univariate	0.281 (0.211-0.350)	<0.001
Multivariate	0.231 (0.161-0.301)	<0.001
hs-CRP (mg/L)		
Univariate	1.404 (0.889-1.918)	<0.001
Multivariate	1.070 (0.580-1.561)	<0.001
TG (mmol/L)		
Univariate	1.737 (0.398-3.075)	0.011
Multivariate	1.011 (-0.202-2.223)	0.102
LDL (mmol/L)		
Univariate	1.221 (-2.034-4.476)	0.461
Multivariate	0.743 (-2.162-3.647)	0.615
Apo-A (g/L)		
Univariate	-35.209 (-61.870 to -8.547)	0.010
Multivariate	-32.342 (-56.475 to -8.210)	0.009

### 3.6 ROC curve analysis of Lp-PLA_2_, hs-CRP, and their combination for diagnostic discrimination of moderate-to-severe coronary stenosis

#### 3.6.1 Diagnostic performance for CHD (CHD vs. control, n = 438)

**Figure 2:**
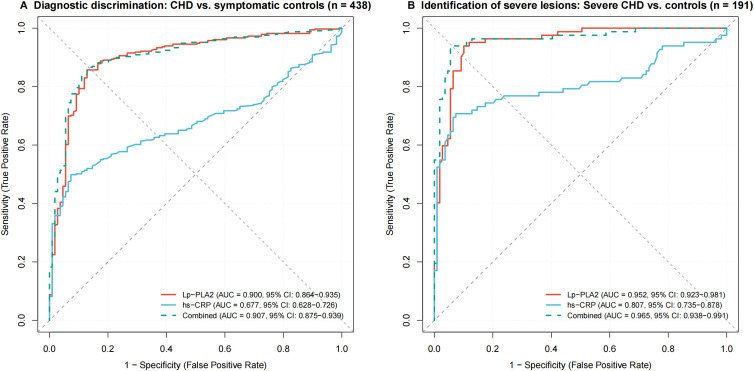
Receiver operating characteristic (ROC) curves for Lp-PLA_2_, hs-CRP, and combined biomarker model. **(A)** Diagnostic discrimination between coronary heart disease (CHD) patients and symptomatic controls (n = 438). **(B)** Identification of severe coronary lesions (Gensini score >56) versus symptomatic controls (n = 191). AUC, area under the curve.

The discriminatory ability of Lp-PLA_2_, hs-CRP, and their combination for distinguishing CHD from controls was evaluated using ROC curve analysis. Lp-PLA_2_ yielded an AUC of 0.900 (95% CI: 0.864-0.935, p < 0.001), with an optimal cutoff value of 127 ng/mL, sensitivity of 85.7%, and specificity of 87.2%. hs-CRP showed an AUC of 0.677 (95% CI: 0.628–0.726, p < 0.001), with an optimal cutoff value of 3.51 mg/L, sensitivity of 49.8%, and specificity of 92.7%. The combined model yielded an AUC of 0.907 (95% CI: 0.875-0.939, p < 0.001), sensitivity of 86.3%, and specificity of 87.2%. The combined model demonstrated significant incremental diagnostic value over hs-CRP alone (ΔAUC = 0.230, Z = 12.412, p < 0.001), though the improvement over Lp-PLA_2_ alone was modest and did not reach statistical significance (ΔAUC = 0.007, Z = 0.794, p = 0.427) (**Figure 2A**).

#### 3.6.2 Identification of severe lesions (severe CHD vs. control, n = 191)

In the secondary analysis restricted to severe CHD (Gensini >56) versus controls, Lp-PLA_2_ achieved an AUC of 0.952 (95% CI: 0.923–0.981, p < 0.001), hs-CRP achieved 0.807 (95% CI: 0.735–0.878, p < 0.001), and the combined model achieved 0.965 (95% CI: 0.938–0.991, p < 0.001), with sensitivity of 93.9% and specificity of 94.5%. (**Table 7**, **Figure 2B**).

**Table 7:** Receiver operating characteristic curve analysis of Lp-PLA_2_, hs-CRP, and their combination for CHD diagnosis and severe lesion identification.

Analysis	Variable	AUC	*P*-Value	AUC95% CI	Cut-off	Sens	Spec
CHD vs. Control (n=438)	Lp-PLA_2_ (ng/mL)	0.900	<0.001	0.864-0.935	127 ng/mL	0.857	0.872
	hs-CRP (mg/L)	0.677	<0.001	0.628-0.726	3.51 mg/L	0.498	0.927
	Combined Diagnosis	0.907	<0.001	0.875-0.939	0.676	0.863	0.872
Severe CHD vs. Control (n=191)	Lp-PLA_2_ (ng/mL)	0.952	<0.001	0.923-0.981	140.4 ng/mL	0.939	0.899
	hs-CRP (mg/L)	0.807	<0.001	0.735-0.878	3.51 mg/L	0.707	0.927
	Combined Diagnosis	0.965	<0.001	0.938-0.991	0.513	0.939	0.945

AUC, area under the curve; CI, confidence interval; Sens, sensitivity; Spec, specificity. Optimal cutoffs were determined independently for each analysis using the Youden index. The two analyses address distinct clinical questions and therefore have different optimal cutoffs.

## 4 Discussion

### 4.1 Study perspective

Despite a gradual decline in CHD mortality in recent years, the incidence of CHD among younger and middle-aged populations has continued to rise (Xie et al., 2025). Together with population aging, this trend has led to a growing healthcare burden and significant socioeconomic impact. The early manifestations of CHD are often insidious, and without timely intervention, disease progression may result in severe cardiovascular events (Agamy et al., 2025). Consequently, the development of accurate lesion assessment tools for the early identification and stratification of coronary lesions is critical for optimizing clinical decision-making and improving patient outcomes. Inflammatory biomarkers such as Lp-PLA_2_ and hs-CRP have received increasing attention for their potential utility in evaluating cardiovascular risk. However, the majority of existing studies have concentrated on the diagnostic or prognostic value of individual biomarkers, whereas investigations addressing the diagnostic value of their combined use and their association with coronary lesion severity remain limited. The value of these biomarker findings lies in characterizing cross-sectional associations with coronary lesion severity in symptomatic patients undergoing coronary angiography. These exploratory results may inform future prospective studies evaluating whether combined biomarker assessment could complement conventional clinical evaluation in similar patient populations.

The present study aimed to evaluate the clinical value of combined Lp-PLA_2_ and hs-CRP measurement in individuals undergoing coronary angiography. The findings demonstrated that: (1) Lp-PLA_2_ and hs-CRP are independent risk factors for CHD; and (2) both biomarkers are significantly associated with lesion severity. Notably, Lp-PLA_2_ demonstrated excellent diagnostic discrimination for CHD (AUC = 0.900) and severe lesions (AUC = 0.952), while the incremental value of hs-CRP when combined with Lp-PLA_2_ was modest and did not reach statistical significance in DeLong’s test.

### 4.2 Mechanism and biomarkers

The development of CHD is multifactorial, involving complex interactions among genetic predisposition, environmental exposures, and modifiable lifestyle factors. Traditional risk factors that have been consistently confirmed include advanced age, smoking, diabetes, hypertension, and dyslipidemia (Regmi and Siccardi, 2023). In the present study, age, smoking, and a history of diabetes were significantly more prevalent risk factors in the CHD group compared to the control group and remained independently associated with the presence of coronary lesions after adjustment for confounding variables. These findings are consistent with previous evidence. No significant associations were observed between a history of hypertension or drinking and CHD, which may be attributable to sample-specific characteristics or the influence of potential confounders. Although not the primary focus of this investigation, the importance of glycemic control and early smoking cessation should be emphasized in routine clinical practice, given their well-established roles in reducing cardiovascular risk. These modifiable factors remain critical targets for both primary prevention and secondary intervention.

The observed associations between serological biomarkers and coronary AS in this study may be primarily explained by two key mechanisms:

1. Lipid Metabolism: Dyslipidemia is a well-established risk factor for AS. The prevalence of dyslipidemia in the Chinese population has increased in recent years. In the multivariate model, LDL-C remained a strong independent predictor of CHD (OR = 4.239, 95% CI: 2.409-7.460, p < 0.001), consistent with its established role as a pathogenic risk factor for ASCVD according to the *Chinese Guidelines for Lipid Management (Primary Care, 2024 edition)*, LDL-C is a pathogenic risk factor for atherosclerotic CVD (ASCVD) (Wang et al., 2024). Under physiological conditions, LDL-C concentrations are generally maintained at 0.5–1.0 mmol/L (approximately 20–40 mg/dL), within which the risk of AS remains relatively low. When LDL-C exceeds this range, its accumulation in the arterial intima increases substantially, thereby accelerating the development of AS. A reduction of 1 mmol/L in LDL-C decreases the risk of acute vascular events by 20–25% (Ference et al., 2017; Grundy et al., 2019; Mach et al., 2020).

HDL-C levels varied across severity groups (**Table 1**), with the highest levels observed in the moderate lesion group. While HDL-C showed a weak positive correlation with the Gensini score (r = 0.111, *p* = 0.044), this relationship is likely confounded by acute-phase lipid alterations in AMI patients and metabolic interdependence with triglycerides (r = 0.460, *p* < 0.001). These findings underscore that HDL-C biology in acute coronary settings is complex and may not follow a simple linear pattern. Future prospective studies with standardized lipid assessment protocols are warranted.

TC and TG, both routinely measured lipid parameters, also play important roles in the progression of CVD. Elevated TC and TG levels significantly increase the risk of AS, promote plaque formation, narrow the vascular lumen, impair blood flow, and consequently elevate the risk of CHD, myocardial infarction, and stroke (Jung et al., 2022).

Lp(a), a lipoprotein structurally similar to LDL-C, has been confirmed by epidemiological and genetic studies as an independent pathogenic risk factor for CVD, with levels largely determined by genetic factors (Kronenberg et al., 2022a; Kronenberg et al., 2022b; Reyes-Soffer et al., 2022). Lp(a) may accelerate plaque progression and increase the risk of cardiovascular events through the promotion of AS, thrombosis, and inflammatory responses. In this study, Lp(a) levels were significantly higher in the CHD group compared to the control group (*p* < 0.05), consistent with previous findings. However, no significant differences were observed among CHD subgroups stratified by lesion type, number of diseased vessels, or lesion severity. Previous work by Shapiro et al. emphasized the importance of Lp(a) measurement in adults with a personal or family history of early-onset ASCVD and recommended that such individuals undergo at least one lifetime measurement, along with early lifestyle interventions to reduce cardiovascular risk (Nurmohamed et al., 2022; Shapiro et al., 2025).

2. Inflammatory Response: Inflammation is a key mechanism in the initiation and progression of AS. Lp-PLA_2_, a member of the phospholipase A_2_ family, is primarily secreted by inflammatory cells like macrophages and monocytes, and its plasma levels increase significantly in response to inflammatory stimulation (Balsinde et al., 2002; Huang et al., 2020). Approximately 70% of circulating Lp-PLA_2_ is bound to LDL-C, while the remainder is associated with HDL-C (Mouchlis et al., 2022). Lp-PLA_2_ catalyzes the hydrolysis of LDL-C phospholipids, producing lysophosphatidylcholine (Lyso-PC) and oxidized free fatty acids (oxFFA), which act as potent chemoattractants for monocytes and leukocytes, facilitating their recruitment to the vascular intima and initiating inflammatory cascades (Maiolino, 2015).

Lyso-PC and oxFFA are subsequently internalized by macrophages via scavenger receptors, promoting foam cell formation and lipid accumulation, hallmarks of early atherosclerotic plaque development (Silva et al., 2011; Lara-Guzmán et al., 2018). Lyso-PC enhances the generation of reactive oxygen species (ROS), further amplifying oxidative stress. Through these pro-inflammatory and pro-oxidative mechanisms, Lp-PLA_2_ actively contributes to the progression of AS (Bargieł et al., 2021).

hs-CRP is another key biomarker of systemic inflammation and has been consistently studied in the context of CVD. A meta-analysis reported that individuals in the highest quartile of hs-CRP levels had a 1.5-fold increased risk of major cardiovascular events compared with those in the lowest quartile even after adjustment for traditional risk factors (odd ratio = 1.5) (Kamath et al., 2015). In addition, elevated hs-CRP has been associated with an increased risk of in-stent restenosis (ISR) following percutaneous coronary intervention in patients with CHD, and with poorer long-term prognosis, indicating its utility as a predictive marker for ISR outcomes (Zhu et al., 2018). A prospective study further indicated that incorporating hs-CRP into vascular risk assessments for patients with type 2 diabetes mellitus improved the prediction of both macrovascular and microvascular complications (Aryan et al., 2018).

In the present study, patients in the CHD group exhibited significantly higher levels of hs-CRP and Lp-PLA_2_ compared with those in the control group, further supporting the involvement of these biomarkers in the occurrence and progression of CHD. These findings are consistent with existing theories regarding the role of inflammatory responses in AS. The results reinforce the utility of Lp-PLA_2_ and hs-CRP as valuable biomarkers for cardiovascular risk assessment, highlighting their clinical relevance in early detection and risk stratification. Collectively, these findings provide a foundation for the development of precision strategies aimed at the prevention and management of CHD.

### 4.3 Correlation of Lp-PLA_2_, hs-CRP, and their combination with CHD

In this study, both hs-CRP and Lp-PLA_2_ were identified as independent risk factors for the development of CHD in the multivariate model. To evaluate their diagnostic utility, we performed two ROC analyses addressing distinct clinical questions.

Diagnostic discrimination for CHD versus symptomatic controls. Lp-PLA_2_ demonstrated excellent discriminatory performance, substantially outperforming hs-CRP. DeLong’s test indicated that the AUC difference between the combined model and Lp-PLA_2_ alone was not statistically significant (ΔAUC = 0.007, Z = 0.794, p = 0.427), suggesting that Lp-PLA_2_ is the primary driver of diagnostic accuracy and that hs-CRP contributes minimal incremental value for CHD detection in this cohort. In contrast, the combined model significantly outperformed hs-CRP alone (ΔAUC = 0.230, Z = 12.412, p < 0.001).

Identification of severe coronary lesions (severe CHD vs. control, n = 191). The incremental gain of the combined model over Lp-PLA_2_ alone was modest (ΔAUC = 0.012, Z = 1.137, p = 0.256), whereas the improvement over hs-CRP alone was substantial (ΔAUC = 0.257, Z = 6.863, p < 0.001).

These findings indicate that Lp-PLA_2_ is a robust standalone biomarker for both CHD diagnosis and severe lesion identification, with hs-CRP providing limited additional discriminatory value when combined with Lp-PLA_2_. The interpretive value of the combined model may therefore lie not in improving discriminatory accuracy beyond Lp-PLA_2_ alone, but in offering complementary information regarding inflammatory status, which may inform pathophysiological characterization and future risk stratification research.

To further examine the association between these biomarkers and disease severity, subgroup analyses were performed. Stratification by the number of diseased vessels indicated that Lp-PLA_2_ levels increased progressively (three-vessel > double-vessel > single-vessel), while hs-CRP levels were significantly higher in the three-vessel and double-vessel groups compared with the single-vessel group. These results are consistent with previous studies (Lao et al., 2023; Yang et al., 2024; Zhong et al., 2024), supporting a positive correlation between biomarker levels and the extent of coronary artery involvement.

Analysis based on clinical classification demonstrated that patients with AMI had significantly higher Lp-PLA_2_ and hs-CRP levels than those with UAP, while patients with UAP had higher levels than those with SAP. The pronounced elevation of hs-CRP in AMI patients is consistent with its role as an acute-phase reactant, which increases rapidly following myocardial necrosis. However, sensitivity analyses excluding AMI patients confirmed that hs-CRP remained significantly associated with CHD in the stable and unstable angina cohort, suggesting that chronic low-grade inflammation also contributes to its elevation across the spectrum of coronary disease.

In summary, Lp-PLA_2_ and hs-CRP reflect the status of coronary artery lesions through pathways involving inflammation and oxidative stress. Their measurement is simple, cost-effective, and reproducible, and therefore indicates potential for clinical application. While combined detection did not significantly improve AUC beyond Lp-PLA_2_ alone for either diagnostic or severity stratification purposes, the dual-marker approach may still offer clinical value by providing complementary pathophysiological information: Lp-PLA_2_ primarily reflects vascular-specific inflammatory and oxidative activity, whereas hs-CRP indicates systemic inflammation. Statins, like atorvastatin, have been reported to significantly reduce Lp-PLA_2_ and hs-CRP levels, potentially lowering the risk of cardiovascular events through lipid-lowering, anti-inflammatory effects, and plaque stabilization (Mourouzis et al., 2021; Rahman et al., 2023). Regular monitoring of these biomarkers may warrant further investigation in prospective cohorts to evaluate their potential role in identifying high-risk phenotypes and informing treatment response monitoring, thereby providing a basis for future research on preventive strategies in CHD.

### 4.4 Innovations and limitations

#### 4.4.1 Innovations

This study systematically examined the combined application of Lp-PLA_2_ and hs-CRP in the assessment of coronary artery lesions. Prior studies have primarily focused on the relationship between a single biomarker and lesion severity. Although hs-CRP, as a marker of systemic inflammation, has been demonstrated to be independently and positively correlated with lesion severity, and Lp-PLA_2_, as a key enzyme involved in lipid metabolism and vascular inflammation, has been associated with plaque stability, the use of individual biomarkers provides only a partial view of the complex pathophysiology of CHD. By integrating both biomarkers, this study provided a more comprehensive evaluation of the pathophysiological processes underlying vascular injury.

#### 4.4.2 Limitations

This study has several limitations. First, its single-center, retrospective design and limited sample size may restrict generalizability; prospective multicenter studies are warranted. Second, the absence of long-term follow-up precludes evaluation of biomarker predictive value for future cardiovascular events. Third, hs-CRP was measured at admission, which in AMI patients may capture acute-phase inflammation; serial measurements would better distinguish acute from chronic contributions. Fourth, the significant age difference between groups, though consistent with atherosclerosis epidemiology, represents a case-control matching limitation; age-matched sensitivity analyses confirmed robustness. Fifth, several lipid variables (apoB, apoA, TG, Lp(a), Hcy) were excluded from multivariate models due to quasi-complete separation, which may have omitted potentially relevant biomarkers. Sixth, the ROC analyses were designed to address two distinct clinical questions: (1) diagnostic discrimination of CHD versus symptomatic controls, and (2) identification of severe lesions among patients with confirmed CHD. Future studies should evaluate biomarker performance for severity stratification within larger CHD cohorts with adequate representation across the full spectrum of lesion severity. The unexpected trend of increasing HDL-C with lesion severity (**Table 1**) should be interpreted with caution. While this finding may reflect acute-phase lipid alterations in AMI patients or metabolic interactions with triglycerides, the absence of systematic statin use documentation and the lack of serial lipid measurements preclude definitive conclusions. This observation highlights the complexity of HDL-C biology in acute coronary settings and warrants validation in prospective cohorts with controlled lipid assessment protocols. Seventh, optimal cut-off values were derived internally and require external validation. Finally, formal baseline clinical models and incremental predictive metrics (NRI, IDI) could not be constructed due to sample size constraints.

## 5 Conclusion

Serum levels of Lp-PLA_2_ and hs-CRP were positively correlated with the severity of coronary artery lesions, suggesting their potential utility as auxiliary indicators for assessing the extent of coronary artery disease.

Both Lp-PLA_2_ and hs-CRP were identified as independent risk factors for CHD in this cross-sectional analysis. Lp-PLA_2_ demonstrated excellent standalone discriminatory performance for CHD and severe coronary lesions. While combined detection with hs-CRP did not significantly improve AUC beyond Lp-PLA_2_ alone, the dual-marker approach provides complementary pathophysiological information that may inform future risk stratification research.

## Data Availability

The original contributions presented in the study are included in the article/supplementary material. Further inquiries can be directed to the corresponding author.
